# Waterpipe tobacco smoking in healthcare students in the University of Jordan

**DOI:** 10.3389/fpubh.2025.1576868

**Published:** 2025-04-16

**Authors:** Amjad Bani Hani, Shahd Mansour, Moaath M. Al Smady, Farah Bani Hani, Seba Mohanned Obeidat, Eman Ahmed Zahran, Nour Awamleh, Rama Rayyan, Farah T. Bani-Khaled, Mahmoud Odeh, Lana T. Alshdaifat, Rami Addasi, Raed Al-Taher

**Affiliations:** ^1^Department of General Surgery, The University of Jordan, Amman, Jordan; ^2^School of Medicine, The University of Jordan, Amman, Jordan

**Keywords:** waterpipe tobacco smoking (WTS), healthcare students, prevalence, misinformation, societal acceptance, health risks

## Abstract

**Introduction:**

Waterpipe smoking has gained popularity globally, often perceived as a safer alternative to cigarettes, particularly among young adults. This study aimed to assess the prevalence, socio-demographic effects, knowledge, attitudes, and behaviors toward waterpipe tobacco smoking (WTS) among healthcare students in Jordan.

**Methods:**

Using a quantitative cross-sectional design, data was collected from August 2022 to February 2023 through a structured, anonymous online survey. Out of 2003 responses, 1988 met the inclusion criteria.

**Results:**

The prevalence of waterpipe tobacco smoking was 46.3%, predominantly among males (59.6%). Univariate analysis linked WTS with age, gender, year of study, father’s education, and cigarette smoking. Logistic regression showed higher WTS odds in males and cigarette smokers. Waterpipe tobacco smokers viewed WTS as less addictive than cigarettes.

**Discussion:**

High WTS prevalence among healthcare students suggests societal acceptance and misinformation-driven use, despite awareness of health risks. Comprehensive studies and educational interventions are recommended.

## 1 Introduction

Hookah, Narghile, Shisha, and Hubble bubble, these are all common names used worldwide to describe waterpipe tobacco smoking (WTS), a method of tobacco consumption which originated in India and the Middle East in the 16th century. It was developed by physician Hakim Abul Fath as an allegedly safer alternative to smoking, as it involves the passage of the smoke through water prior to its inhalation which was believed to filter out toxins reducing its harm and addiction (1). However, current research refutes his claims, with various studies linking WTS to numerous health risks such as periodontitis, cardiovascular diseases, metabolic disorders, respiratory illnesses, pregnancy complications, and various cancers (2–4). Additionally, studies indicated up to a tenfold increase in carbon monoxide inhalation due to the long duration of WTS smoking sessions, exposing users to higher level of carcinogens and heavy metals (5, 6).

Despite these risks, this practice has gained popularity worldwide, transcending cultural boundaries to become prevalent in several regions such as Europe, North Africa, and the United States (7). For instance, a study conducted among university students from four major public universities in the Western Cape, across all faculties found that 63% of them had smoked a waterpipe at least once in their lives (8). Similarly, data from the 2011 National Youth Tobacco Survey (NYTS) found that 7.3% of adolescents in the United States reported WTS at least once in their lifetime (9). Additionally, WTS was recorded with a prevalence of 4.8% among international medical students in Germany and Hungary (10). However, despite its growing popularity, the persistent misconception of WTS being a safer alternative continues to drive its use, highlighting the need for better public awareness of its health consequences.

A study completed in King Saud University in 2019 reported several common reasons behind WTS among university students including passing time (45.2%), relieving stress (33.3%), and the availability of various flavors of tobacco which attracts individuals with various taste preferences (11). Another study showed that the increase in WTS prevalence can be attributed to various factors like its easy acceptability, as it is commonly enjoyed in cafes and lounges among friends (7). It is increasing prevalence may be largely attributed to its social allure, as it is often viewed as communal activity (5, 6). Jordan, located in the northern part of the Arabian Peninsula, has a diverse population consisting of both urban and rural communities, with a total population of around 11 million people (12). Its population has a slightly higher proportion of males compared to females, with males comprising 52.9% of the country’s demographic (13). Additionally, Jordan has one of the youngest demographics with 63% of its inhabitants under the age of 30 (14).

Despite the country’s ongoing efforts to reduce tobacco consumption, Jordan has one of the highest rates of tobacco use in the region including cigarettes, waterpipes, cigars and pipes, with a prevalence of 32.3% (15). The country ranks second globally for tobacco smoking among adult males (70.2%), with a notable prevalence of 23.3% among the youth (16, 17). Cigarette smoking is the most prevalent form of tobacco use, accounting for 93.0% of tobacco use, and WTS is the second most common form, representing 8.6% of the cases (15). A recent study in Jordan reported that 41.3% of urban Jordanians over the age of 18 engaged in WTS (18). WTS has experienced a notable rise in several countries in the Middle East such that Turkey recorded 32.7% among university students,29.5% in Lebanon, 51.5% in United Arab Emirates, and Saudi Arabia witnessed an increase from 36.6% in 2010 to 46.6% in 2019 (19). However, Jordan stands out with a particularly high prevalence among its university students, where approximately 56% have used waterpipes (20). This high level of tobacco use has led the United Nations to declare the rising tobacco epidemic in Jordan as a public health emergency.

These findings extend to healthcare students as well, making it crucial to study the underlying reasons as this demographic tends to have a critical role in promoting public health and modeling healthy behavior in their respective community (2). In a study targeting university students in Jordan, students from medical faculties demonstrated more knowledge and awareness about the risks and harmful effects of WTS in comparison to their non-medical peers (3). Despite this awareness, many healthcare students still choose to engage in this activity.

Thus, gaining a comprehensive understanding of the reasoning behind their behavior can provide an insight into potential gaps in medical education and challenges faced by this specific population. This study aims to investigate the prevalence, associated factors, and knowledge of WTS in healthcare students in Jordan.

## 2 Methodology

### 2.1 Study design

This study took place at the healthcare faculties of the University of Jordan (UJ), Jordan’s largest and oldest university, located in the capital city Amman (21). Its central location makes it an attraction to students from various backgrounds across Jordan, with an enrollment of over 38,418 students as of 2023 (22). It comprises six medical faculties, holding the highest number of healthcare students among all other institutions in Jordan (23). This study utilized a quantitative cross-sectional design to investigate the prevalence and associated factors of WTS among healthcare students in the country. The cross-sectional nature of our study enabled us to study multiple outcomes within a single time frame.

### 2.2 Sampling technique

A random probability sampling method was implemented to select a sample from the total population of 11,323 healthcare students currently enrolled in healthcare specialties like medical laboratory sciences, rehabilitation, medicine, dentistry, pharmacy, and nursing programs at the University of Jordan (22). The use of this method ensured a sample that is representative of the broader student population, enhancing the generalizability and statistical validity of our findings. It also contributed to reducing researcher bias, consequently increasing the reliability of our study.

### 2.3 Data collection

The data was collected between August 2022 and February 2023 via a structured, anonymous, self-administered online survey developed using Google forms. This instrument was developed and validated based on The Grounded Psychometric Development and Initial Validation of the Health Literacy Questionnaire (HLQ), as well as three other published studies: *Measuring Waterpipe Tobacco Smoking in Survey Research* by Erin L. Sutfin, *Characteristics, Reasons, Behavior, and Knowledge Toward Waterpipe Smoking in Saudi Arabia* by Elluru Venkatesh, and the *Global Adult Tobacco Survey (GATS)—2020.*It was then modified and adapted to suit the purpose of our study (24–27). The privacy provided by this method of data collection ensured that students felt comfortable disclosing their smoking habits, reducing potential social desirability bias.

The survey consisted of 42 questions, defining WTS as “a form of tobacco consumption that utilizes a single or multi-stemmed instrument to smoke flavored or non-flavored tobacco, where smoke is designed to pass through water or other liquid before reaching the smoker” (28). It covered questions on demographics, behavior, perception, and knowledge of water pipe smoking as well as comparative views with cigarette smoking. For this research, 42 questions were analyzed, and their outcomes were examined thoroughly.

Out of the 2003 responses received, 1988 were included in the final analysis as they fulfilled the inclusion criteria which includes consent and being a healthcare student at the University of Jordan. When analyzing the grade point average (GPA) with WTS, only 1,542 responses were used as 446 responses were excluded due to incomplete responses. Among the 1988 participants, 920 were WT smokers, however, when asked to provide complete details regarding their WTS habits, only 249 provided complete details. The remaining 671 incomplete responses were excluded during the analysis of tendencies, behaviors, beliefs, knowledge and perceptions of WT smokers.

### 2.4 Data analysis

This data was analyzed using IBM SPSS Statistics version 27.0.1. Categorical variables were presented as numbers and percentages, and continuous variables as mean and standard deviation. Univariate analysis was performed to assess associated factors with waterpipe smoking, using independent-sample t-test for continuous variables and Chi-square test for categorical variables. Any *p* value < 0.05 was considered to be significant. Post-hoc analysis was utilized with *p*-value set according to the Bonferroni adjustment (29). Binary logistic regression was then carried out to analyze the predictive variables associated with waterpipe smoking. A goodness-of-fit test was carried out through Hosmer-Lemeshow test, indicating the model as a good fit with a *p*-value higher than 0.05 (*p* = 0.955). The logistic regression test was carried out with the exclusion of GPA due to incomplete responses.

Behavior, perception and knowledge were assessed using a Likert scale of 3 levels (Disagree, Neutral, Agree). The analysis of knowledge and perception’s association with waterpipe smoking was assessed using Chi-square tests.

### 2.5 Ethical considerations

This study received approval from the Institutional Review Board (IRB) of the Ministry of Health (MOH). Additionally, participants were informed of the purpose of the study and were asked to sign their informed consent prior to their participation. They were assured of the confidentiality and anonymity of their responses, as well as their right to withdraw at any time.

## 3 Results

### 3.1 Participant characteristics

The total number of respondents in this study was 1988. Over half of the participants (65.7%) were female. Two-thirds of the participants were enrolled in medicine (61.1%). The majority of students were fourth and fifth-year students (23.2 and 22.9%, respectively) as medicine in Jordan is a six-year degree. Both father’s and mother’s education of the participants were mainly bachelor’s degrees (52.2 and 63.6%, respectively). The mean age of our sample was 21.26 years (SD = 2.86), as post-graduate students in the healthcare field enrolled in further programs (internship, residency, fellowship, masters, etc.) were included in our sample. GPAs were averaged at 3.33/ 4.0 (SD = 0.43). Out of our sample, 13.8% (*n* = 274) reported smoking cigarettes (Table 1).

**Table 1:** Demographics and Their Univariate Analysis Regarding Association with WTS

Variables		All participants (n=1988)	Smokes Water-pipe		*P*-value
			No	Yes	
Age, mean (SD)		21.26 (2.86)	20.99 (2.79)	21.58 (2.92)	**<0.001**
GPA, mean (SD)^a^		3.33 (0.43)	3.36 (0.44)	3.30 (0.41)	**0.008**
Gender, n (%)	Female	1306 (65.7%)	791 (60.6%)	515 (39.4%)	**<0.001**
	Male	682 (34.3%)	277 (40.6%)	405 (59.4%)	
College, n (%)	Medicine	1215 (61.1%)	654 (53.8%)	561 (46.2%)	0.731
	Dentistry	246 (12.4%)	128 (52.0%)	118 (48.0%)	
	Pharmacy/Doctor of Pharmacy	215 (10.8%)	116 (54.0%)	99 (46.0%)	
	Rehabilitation	94 (4.7%)	50 (53.2%)	44 (46.8%)	
	Nursing	182 (9.2%)	96 (52.7%)	86 (47.3%)	
	Medical Laboratory	36 (1.8%)	24 (66.7%)	12 (33.3%)	
Year of Study, n (%)	1st Year Student	243 (12.2%)	165 (67.9%)	78 (32.1%)	**<0.001**
	2nd Year Student	308 (15.5%)	182 (59.1%)	126 (40.9%)	
	3rd Year Student	343 (17.3%)	179 (52.2%)	164 (47.8%)	
	4th Year Student	462 (23.2%)	234 (50.6%)	228 (49.4%)	
	5th Year Student	456 (22.9%)	223 (48.9%)	233 (51.1%)	
	6th Year Student	124 (6.2%)	56 (45.2%)	68 (54.8%)	
	Post-graduate	52 (2.6%)	29 (55.8%)	23 (44.2%)	
Father's Education, n (%)	Illiterate	12 (0.6%)	5 (41.7%)	7 (58.3%)	**0.008**
	Primary School	52 (2.6%)	27 (51.9%)	25 (48.1%)	
	Secondary School	269 (13.5%)	163 (60.6%)	106 (39.4%)	
	Bachelors	1038 (52.2%)	574 (55.3%)	464 (44.7%)	
	Post-College Degree	617 (31.0%)	299 (48.5%)	318 (51.5%)	
Mother's Education, n (%)	Illiterate	23 (1.2%)	12 (52.2%)	11 (47.8%)	0.073
	Primary School	60 (3.0%)	28 (46.7%)	32 (53.3%)	
	Secondary School	326 (16.4%)	194 (59.5%)	132 (40.5%)	
	Bachelors	1264 (63.6%)	680 (53.8%)	584 (46.2%)	
	Post-College Degree	315 (15.8%)	164 (48.9%)	161 (51.1%)	
Living Arrangement, n (%)	Lives alone	193 (9.7%)	95 (49.2%)	98 (50.8%)	0.201
	Lives in a dorm/with roommates	121 (6.1%)	72 (59.5%)	49 (40.5%)	
	Lives with family	1674 (84.2%)	901 (53.8%)	773 (46.2%)	
Smokes Cigarettes, n (%)	No	1714 (86.2%)	1043 (60.9%)	671 (39.1%)	**<0.001**
	Yes	274 (13.8%)	25 (9.1%)	249 (90.9%)	

‘All Participants’ are presented as column percentages, while ‘Smokes Waterpipe’ are presented as row percentages.

^a Data was calculated with n = 1542 due to incomplete responses. Bold values represent a significant p-value.^

### 3.2 The association between demographic factors and water-pipe tobacco smoking

Out of our participants, 920 (46.3%), answered ‘Yes’ to WTS. We found that 59.6% of males and 39.4% of females were self-reported WT smokers. Dentistry students had the highest percentage of WT smokers (48.0%), while medical laboratory students had the lowest (33.3%). The average age of WT smokers was higher than non-smokers (*μ* = 21.58, SD = 2.92 vs. *μ* = 20.99, SD = 2.79, respectively). The average GPA of WT smokers was lower than non-smokers (*μ* = 3.30, SD = 0.41 vs. *μ* = 3.36, SD = 0.44). Sixth-year students had the highest percentage of WT smokers (54.8%), while first-year students had the lowest (32.1%). Students with illiterate fathers or mothers holding a primary school diploma had the highest percentage of WT smokers (58.3 and 53.3%, respectively). Of students who lived alone, 50.8% were WT smokers. As for participants who answered ‘No’ for cigarette smoking, 39.1% reported as WT smokers, whereas almost all (90.88%) cigarette smokers were WT smokers (Table 1).

An independent-samples *t*-test revealed statistical significance in mean age (t (1986) = 4.592, *p* = <0.001, 95% CI [0.337, 0.839]) and GPA (t (1540) = −2.655, *p* = 0.008, 95% CI [−0.10025, −0.01506]) between WT smokers and non-smokers. Chi-square test also showed statistical significance for gender X^2^ (1, *N* = 1988) = 71.731, *p* < 0.001, year of study X^2^ (6, *N* = 1988) = 33.303, *p* < 0.001, father’s education X^2^ (4, *N* = 1988) = 13.789, *p* = 0.008, and cigarette smoking X^2^ (1, *N* = 1988) = 254.253, *p* < 0.001 between WT smokers and non-smokers. *Post-hoc* analysis comparing z-scores of two proportions with Bonferroni adjustment was carried out. The proportion of first-year student non-smokers was statistically higher than that of WT smokers (*p* < 0.001). *Post-hoc* analysis also revealed statistical significance of WT smokers with fathers of higher studies education (*p* = 0.01).

Binary logistic regression showed that gender and cigarette smoking were significantly associated with WTS while age, year of study and father’s education were not. GPA was not included in the model due to missing values. According to the model, the odds of males to WT smoke were 1.56 times higher of those of females. Cigarette smokers’ odds of WTS were 13.14 times higher than those who do not smoke cigarettes (Table 2).

**Table 2:** Predictors of WTS

Variables		Odds Ratio	95% C.I.		*P*-value
			Lower	Upper	
Age		1.017	0.973	1.062	0.463
Gender	Female	Ref			
	Male	1.556	1.264	1.915	**<0.001**
Smokes Cigarettes	No	Ref			
	Yes	13.144	8.551	20.203	**<0.001**

Bold values represent a significant *p*-value.

**Figure 1:**
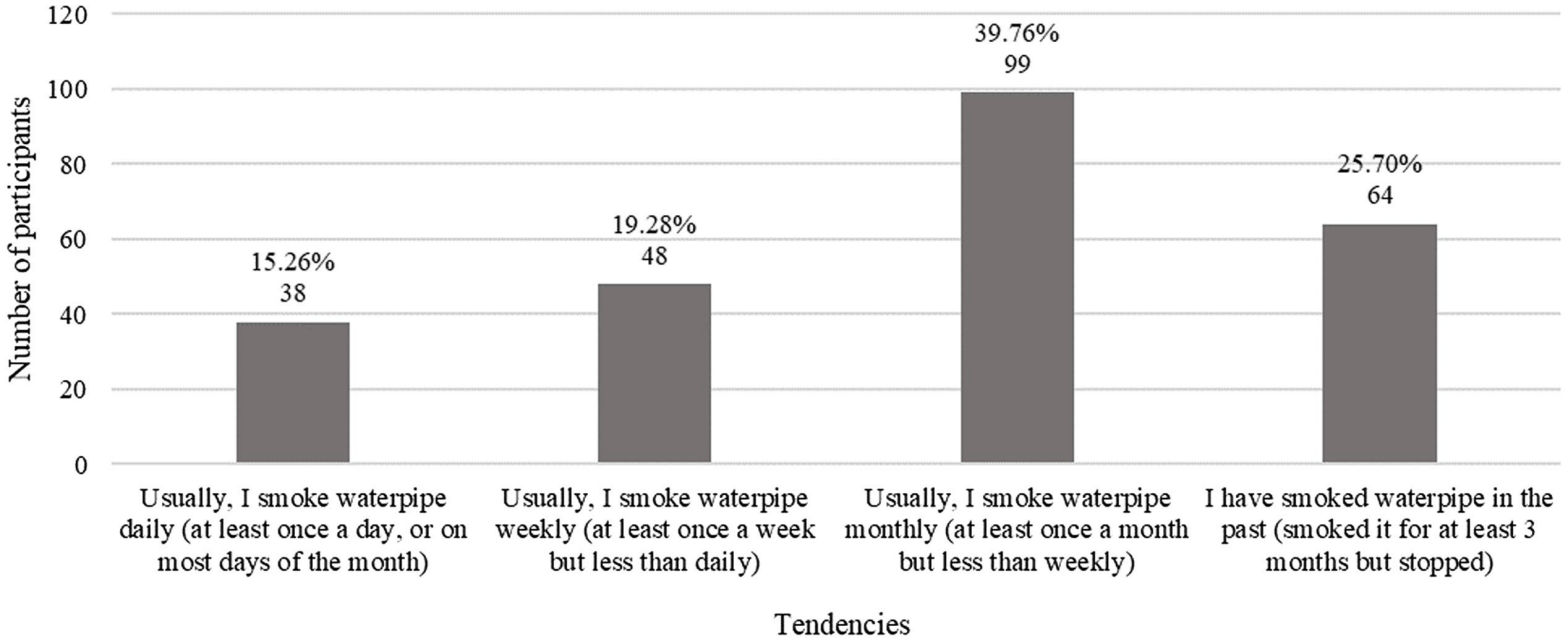
Participants describe their WTS tendencies.

Of the 920 participants who reported WTS, 249 submitted complete responses surrounding their WTS details. The mean age of starting WTS was approximately 17.17 (SD = 2.59; Table 3). More than a third (39.76%, *n* = 99) of WTS participants reported smoking monthly (Figure 1).

**Table 3:** Behavior of WT Smokers

Questions regarding behavior		(*n* = 249)	
Age of Starting WTS, mean (SD)		17.17	(2.59)
Do You Share Your Waterpipe? n (%)	Yes	187	(75.1%)
	No	62	(24.9%)
Can You Quit WTS at Any Time? n (%)	Yes	223	(89.6%)
	No	26	(10.4%)
Do You Intend to Quit WTS? n (%)	Not at all	74	(29.7%)
	In the next month	60	(24.1%)
	In the next 6 months	10	(4.0%)
	In the future	105	(42.2%)
Who Did You First WTS With? n (%)	No one, I was alone	21	(8.4%)
	With one friend	56	(22.5%)
	With more than one friend	121	(48.6%)
	With more than one family member	18	(7.2%)
	With a family member	33	(13.3%)
Where Did You First WTS? n (%)	In a cafe or restaurant	125	(50.2%)
	At a family member's home	22	(8.8%)
	In my own home (apartment, condominium, house)	40	(16.1%)
	At a friend's or acquaintance's home	52	(20.9%)
	In my own dormitory room	10	(4.0%)
How Did Covid-19 Lockdown Affect Your WTS? n (%)	Smoked less during lockdown	85	(34.1%)
	Did not affect it	114	(45.8%)
	I started WTS during lockdown	15	(6.0%)
	I smoked more during lockdown	35	(14.1%)
WTS is Consuming the Following Percentage of My Allowance, n (%)	up to 5%	137	(55.0%)
	up to 25%	35	(14.1%)
	up to 50%	13	(5.2%)
	up to 10%	48	(19.3%)
	more than 50%	16	(6.4%)

Almost all (89.6%) of WT smokers believed they could quit WTS at any time, however 29.7% answered that they do not intend on quitting and 42.2% said they would quit in the foreseeable future. Almost half (48.6%) of WT smokers had their first WTS experience with more than one friend, and 50.2% first WT smoked at cafes or restaurants. Around three-quarters of the WT smoker participants (75.1%) have shared their waterpipe. More than half (55.2%) reported that WTS consumed only up to 5% of their allowance. When asked about the effect of Covid-19 lockdown on their WTS habits, 45.8% of WT smokers said it had no effect at all (Table 3).

Almost half (44.2%) of our WT smoker participants disagreed with WTS being a financial burden. Additionally, 35.3% agreed with sentiment that their parents would object to WTS compared to cigarette smoking, and 27.7% felt neutral about it. WTS gives happiness to around 40.2% of smokers, while 31.8% disagreed with the statement. Almost half (43.8%) disagreed about WTS helping them deal with pressure, as well as helping them fit in at parties or gatherings (53.0%). A large percentage (80.3%) of WT smokers reported that WTS does not improve their image among their friends. Around three-quarters (71.9%) believed that WTS does not improve their academic performance. Lastly, more than two-thirds (67.8%) WT smoke to simply have fun (Table 4).

**Table 4:** Beliefs of WT smokers regarding WTS

Statements regarding beliefs	Disagree	Neutral	Agree
WTS is a Financial Burden to Me	44.2%	30.1%	25.7%
My Parents Wouldn’t Object to WTS Compared to Cigarettes	35.3%	27.7%	37%
WTS Gives Me Pleasure and Happiness	31.8%	28.0%	40.2%
WTS Helps Me Deal with Pressure	43.8%	20.9%	35.3%
WTS Helps Me Fit in at Parties or Gatherings	53.0%	23.7%	23.3%
WTS Improves My Image Among My Friends	80.3%	13.7%	6.0%
WTS Improves My Academic Performance	71.9%	15.7%	12.4%
I WT Smoke to Have Fun	10.9%	21.3%	67.8%

**Figure 2:**
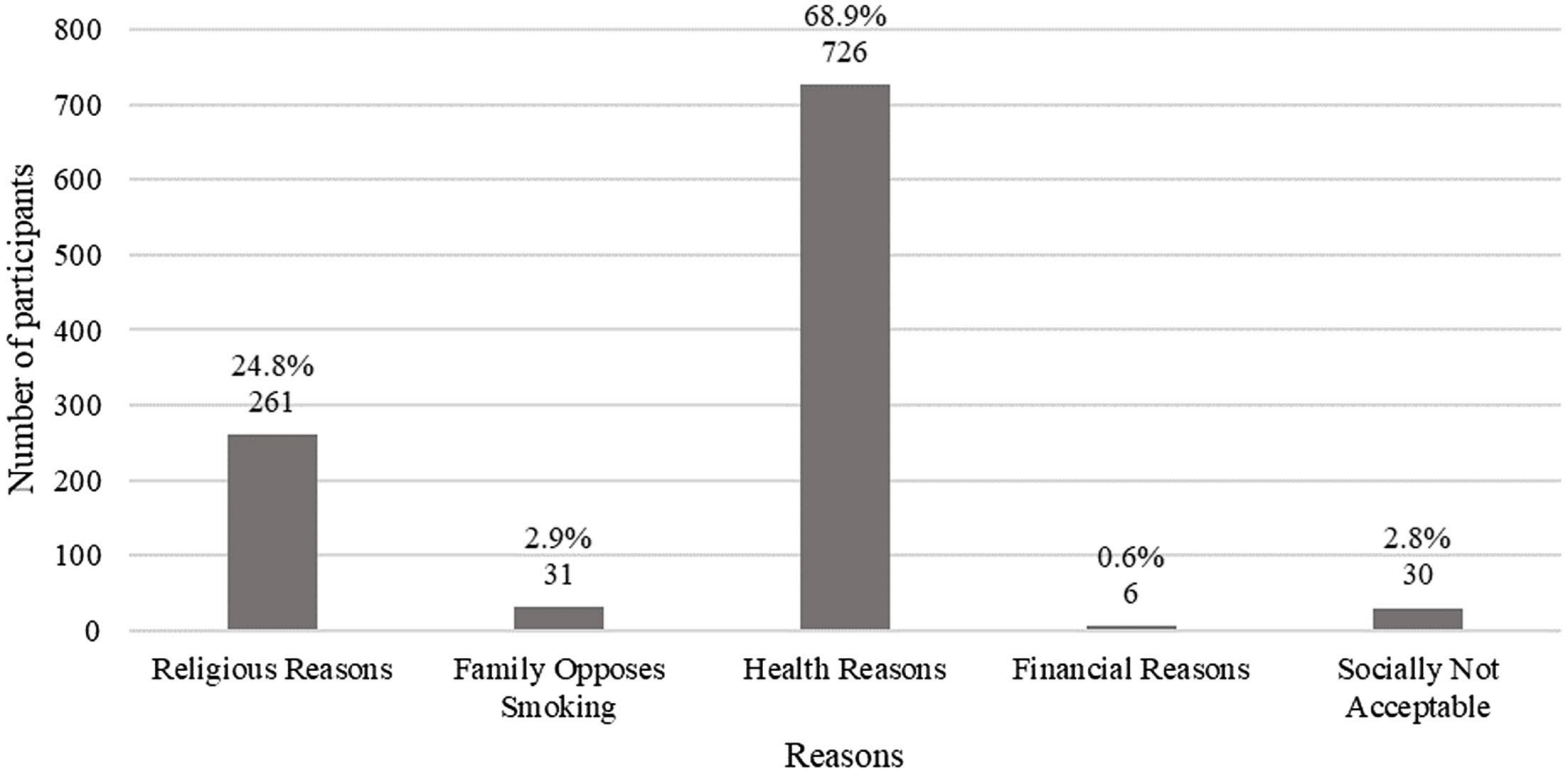
Reasons for not WTS. *n* = 1054 due to missing values.

Of the 1988 participants, 1,068 (53.7%) answered ‘No’ to ever WTS. Over two-thirds (68.88%, *n* = 726) of non-smokers chose health as a reason for abstaining from WTS, with religious reasons following at 24.76% (*n* = 261; Figure 2).

### 3.3 Knowledge and perception of participants regarding WTS

Of our 1988 participants, 59.8% believe that WTS is acceptable to society compared to cigarette smoking. When asked if WTS contains tobacco, 65.2% agreed while the rest disagreed or were neutral regarding the statement. Moreover, 41.9% disagree that WTS helps a person feel more comfortable at a social gathering and 23.7% took a neutral stance. More than three-quarters (77.7%) disagreed with the idea of WTS making a person look more mature. Almost all participants (87.8%) do not believe that WTS is safe due to the water filtering the smoke. Of our participants, 83.2% agreed that WTS causes cardiac diseases, 81.6% agreed that it causes cancer, and 89.0% agreed that it causes respiratory difficulties. As for safety of WTS during pregnancy, 88.4% disagreed. When asked if WTS was easily accessible, 73.8% agreed. Almost half (43.2%) agreed that WTS is accepted by others, while 29.3% disagreed. Almost half (47.0%) of our participants disagreed with the statement that WTS is less addictive that cigarette smoking. Although 41.1% believe it is less harmful than cigarettes, 40.8% disagreed (Table 5).

**Table 5:** Knowledge and Perception of Participants Regarding WTS

Statements regarding knowledge and perception	Disagree	Neutral	Agree
WTS is Acceptable to Society Compared to Cigarettes	16.4%	23.8%	59.8%
Waterpipes Traditionally Contain Tobacco	10.8%	23.9%	65.2%
WTS Helps a Person Feel More Comfortable at a Social Gathering	41.9%	23.7%	34.3%
WTS Makes a Person Look More Mature	77.7%	12.2%	10.1%
WTS is Safe Due to Water Filtering the Smoke	87.8%	7.4%	4.8%
WTS Causes Heart Diseases	4.7%	12.1%	83.2%
WTS Causes Cancer	5.0%	13.5%	81.6%
WTS Causes Respiratory Tract Diseases	3.6%	7.5%	89.0%
WTS is Safe During Pregnancy	88.4%	6.0%	5.5%
Waterpipes Are Easily Available	8.6%	17.6%	73.8%
Waterpipe Smokers Are Accepted by Others	29.3%	27.5%	43.6%
WTS is Less Addictive than Cigarettes	47%	27.6%	25.5%
WTS is Less Harmful Compared to Cigarettes	40.8%	18.1%	41.1%

Chi-square tests revealed statistical difference between WT smokers and non-smoker’s knowledge and perception regarding: WTS acceptability in society (*p* < 0.001), WTS containing tobacco (*p* < 0.001), helping a person feel more comfortable at a social gathering (*p* < 0.001), availability of WTS (*p* = 0.001), acceptability by others (*p* < 0.001), and lower addiction than cigarettes (*p* < 0.001; Table 6).

**Table 6:** Analysis of Knowledge and Perception of WTS Between WT Smokers and Non-Smokers

Statements regarding knowledge and perception	Smokes Water-Pipe Tobacco						P-Value
	No			Yes			
	Disagree	Neutral	Agree	Disagree	Neutral	Agree	
WTS is Acceptable to Society Compared to Cigarettes	20.7%	24.0%	55.2%	11.0%	23.7%	65.3%	**<0.001**
Waterpipes Traditionally Contain Tobacco	10.9%	27.4%	61.6%	10.8%	20.1%	69.1%	**<0.001**
WTS Helps a Person Feel More Comfortable at a Social Gathering	47.4%	23.1%	29.5%	35.4%	24.4%	40.2%	**<0.001**
WTS Makes a Person Look More Mature	79.5%	10.5%	10.0%	75.7%	13.9%	10.3%	0.060
WTS is Safe Due to Water Filtering the Smoke	88.0%	7.1%	4.9%	87.6%	7.7%	4.7%	0.840
WTS Causes Heart Diseases	4.8%	12.2%	83.0%	4.6%	12.0%	83.5%	0.959
WTS Causes Cancer	4.8%	13.6%	81.6%	5.1%	13.5%	81.4%	0.951
WTS Causes Respiratory Tract Diseases	4.0%	8.1%	87.9%	3.0%	6.7%	90.2%	0.264
WTS is Safe During Pregnancy	88.3%	6.7%	4.9%	88.5%	5.1%	6.3%	0.148
Waterpipes Are Easily Available	10.0%	19.3%	70.6%	6.8%	15.5%	77.7%	**0.001**
Waterpipe Smokers Are Accepted by Others	32.4%	28.5%	39.1%	25.6%	26.1%	48.2%	**<0.001**
WTS is Less Addictive than Cigarettes	54.4%	28.5%	17.2%	38.4%	26.3%	35.3%	**<0.001**
WTS is Less Harmful Compared to Cigarettes	41.6%	17.3%	41.2%	39.9%	19.3%	40.8%	0.483

Note: Some questions had approximately 5-15 missing values out of 1068 non-smokers. Bold values represent a significant *p*-value.

## 4 Discussion

Our study examined the prevalence of WTS among healthcare students in Jordan, including demographics, knowledge, attitudes, and behaviors. The findings revealed a high prevalence of WTS at 46.3%, which surpasses the prevalence of cigarette smoking (13.2%). This observation indicates a greater tendency toward WTS compared to cigarettes, thus, highlighting an increased shift in popularity and acceptance toward it among healthcare students. Unlike similar studies conducted in other regions, there is a lack of research that examines WTS prevalence among healthcare students in Jordan, making direct comparisons difficult. However, two other studies reported comparable findings among healthcare students; a WTS prevalence of 37 and 48.9% in Southeastern US and Saudi Arabia, respectively (2, 30).

Age and gender were significantly associated with WTS in our study, with the majority of WT smokers being male (59.6%), despite our sample being predominantly female (65.7%). Similarly, another study with a female dominant sample found that mostly male students WT smoke. The study suggested that social and cultural norms in countries like Saudi Arabia and some Mediterranean countries may discourage female smoking as it is not seen as a socially acceptable behavior (31). This could also explain the results of our study conducted in Jordan. Furthermore, the findings align with Daradka’s study, which also noted the impact of age (32). This may be due to having expanded social networks and increased participation in social events as individuals grow older, as well as greater access to venues offering WTS. In contrast, another study found no significant association of age and gender with waterpipe smoking (19).

Most of our sample of WT smokers stated no significant change in their smoking frequency during Covid-19. This may be due to the participants adhering to a fixed smoking routine that is not affected by the lockdown routine. The finding contradicts that of a study done in Arab countries in 2022, where WTS was significantly higher in Jordan during Covid-19 compared to the WTS levels before the pandemic (33).

Parents’ educational background was observed to play a role as well, with higher levels of paternal education being associated with a higher prevalence. This might be explained by the fact that parents with a higher educational level tend to earn a higher income, enabling their children to afford WTS habits easily. Additionally, the academic year and performance of individuals also contributed significantly, with lower GPAs and higher academic years being associated with higher prevalence. This aligns with Jawad’s study, which found that fifth-year students were more likely to engage in WTS (34). The association between low GPAs and increased prevalence of WTS may be attributed to general unhealthy lifestyle habits that students adopt to manage academic pressure and emotional distress.

A significant correlation was identified between cigarette smoking and WTS, with 90.88% of our cigarette smokers tending to engage in WTS. Another study on Jordanian adults found that participants who smoked cigarettes within the past 30 days were almost 2.69 times more likely to use WTS (35). Similar studies conducted in the US and Lebanon among university students also found cigarette smoking to be a strong predictor of WTS use (36, 37). This may be due to their misconception that other forms of smoking have a lower risk compared to cigarette smoking. Most of our WT smokers reported smoking monthly and 89.6% believed that they can quit at any time, due to possibly considering WTS to be a social activity rather than a habitual act. An intent to quit was noted in 42.2% of the smokers versus 29.7% which stated no intention of quitting. Similar results were seen in a study conducted in Jordan on dental students, where the proportion of WT smokers who had an intent to quit was 42.0% (38). Understanding this perceived sense of self-control over smoking tendencies is crucial to help develop appropriate and effective cessation programs.

As for the non-WT smokers, most cited health reasons, followed by religious reasons as primary motivations for their abstinence. To our knowledge, no previous articles have reviewed motivators for abstaining from WTS, however our findings were similar to those of abstaining from cigarette smoking. For instance, a study conducted among medical students in King Fahad medical city identified these same motives (health and religion) as important markers for not initiating cigarette smoking or giving up on smoking (39).

In addition, most of our waterpipe smokers stated that they only smoke for fun. However, when asked whether it provided them with a sense of happiness and pleasure, the responses were equally divided between agreement and disagreement. This suggests that there may be other underlying reasons behind their decision to WT smoke, and according to our findings, the strong social aspect of WTS might be the motivator. Similar studies have highlighted other reasons beyond fun and enjoyment as motivators for WTS such as peer pressure, relieving social anxiety, for experience and out of curiosity (40).

The majority of our WT smokers stated that they frequently participated in the social aspect of waterpipe sharing. Nearly half of the WT smokers disclosed that they originally initiated this habit with family or friends in public settings like cafes or restaurants and the majority stated that they frequently participated in the social aspect of waterpipe sharing. This finding is consistent with studies that have correlated increased accessibility of waterpipes in cafes and restaurants with higher prevalence rates, as well as studies that have observed its role as a tool for socializing in some communities (19, 41). Contrary to expectations, our sample demonstrated that although WTS was perceived as more socially acceptable than cigarette smoking (59.8%), most participants agreed that it did not make them feel more comfortable at social gatherings.

We observed a disparity between knowledge and behavior among our sample, prompting the need to develop strategies to address these misconceptions urgently. Despite a significant awareness among our population regarding the adverse aspects of WTS such as its tobacco content, addictiveness, and associated health risks like cardiac diseases, cancers, respiratory difficulties, and pregnancy complications, the prevalence of WTS remained to be considerably high. Additionally, responses were varied regarding whether WTS is less harmful than cigarette smoking. These findings align with previous studies that noted students’ general awareness of these risks may not necessarily influence their decision to continue using WTS (3). Regarding the students’ level of knowledge, some studies agreed with our findings while others disagreed (3, 40). This might be due to varying levels of public health among different populations and a lack of unified and effective global efforts to create public awareness which reach their intended populations. As for the evident disparity between understanding the risks but still smoking, there might be an element of optimism bias; where a person thinks they are invulnerable, and a negative event will happen to others but not them. Additionally, students who WTS should be guided to smoking cessation clinics more often, and there is a need for positive role models and continuous educational efforts for them (3, 40).

Our findings highlight the influence of actual experience on the levels of knowledge and perception of WTS. Significant differences were observed between WT smokers and non-smokers in their perception of the acceptability of WTS compared to cigarettes, awareness of the tobacco content in waterpipes, social acceptability, and availability. Additionally, more WT smokers tended to believe that WTS brought them comfort at social gatherings, whereas most non-WT smokers disagreed. Furthermore, non-WT smokers disagreed with WTS being less addictive than cigarette smoking, while WT smokers were evenly split between agreement and disagreement. This contrasts with a study that found that university students who WT smoke perceived WTS as less addictive. This suggests that healthcare students may have a lower level of misinformation compared to other university students (40).

Based on our findings, several actions are needed to reduce waterpipe smoking (WTS) among healthcare students at the University of Jordan and address the underlying causes and behaviors. Smoking cessation programs should target both cigarette and waterpipe smoking, given the direct relationship between the two, with 90.88% of cigarette smokers also engaging in WTS. These programs must focus on combating the belief that WTS is a harmless social activity, while promoting healthy stress-coping strategies and offering a support network for students. In addition, the study highlights the significance of religious and health motivations for non-smokers. Since religion plays a pivotal role in Jordan, religious leaders could be key in promoting anti-smoking messages. Collaborations between religious leaders and universities to host seminars on the religious stance against smoking and its health consequences could align religious values with healthier lifestyle choices. Furthermore, stronger regulations are needed to limit WTS in public spaces. This includes banning its use in settings like cafes and restaurants and enforcing existing laws that prohibit underage purchasing and smoking, with clear and strict penalties for violations. By implementing these strategies, WTS prevalence could be reduced and a healthier environment could be provided for students.

A limitation to this paper is its exclusive focus on healthcare students at the University of Jordan, which might limit its generalizability to students from other universities. However, the effect of this limitation is alleviated, due to utilizing a large sample size from Jordan’s largest university, which holds the highest number of healthcare students. Additionally, the cross-sectional nature of our study limits our ability to establish causality as there may be still some residual confounding factors despite our efforts to control them. In addition to that, our choice of data collection method does not account for recall bias.

To the best of our knowledge, this study is one of the few recent papers exploring WTS among Jordanian healthcare students specifically, as most studies have shifted their focus to e-cigarettes. Despite the growing popularity of e-cigarettes, WTS prevalence continues to rise, which highlights the importance of research and continuous efforts to combat this increase.

A longitudinal study that follows WT smokers, tracking the changes in their behavior over time, and evaluating the long-term effects of cessation programs and educational campaigns is recommended to gain further insight into the factors affecting WTS tendencies in this crucial group, due to their vital societal role and the extensive healthcare-related responsibilities.

## 5 Conclusion

Our findings indicate that the recorded high prevalence of WTS among healthcare students presents a public health concern that is primarily influenced by societal acceptance and misinformation despite the students’ awareness of the associated health risks. Future comprehensive studies on the impact of social approval are suggested. The social nature of WTS introduces significant public health concerns regarding second-hand smoke exposure. In order to develop effective cessation programs, governments must take action to address the persistence of misinformation, as well as work on reducing the accessibility of waterpipes, and banning WTS in public and social venues.

## Data Availability

The original contributions presented in the study are included in the article/supplementary material, further inquiries can be directed to the corresponding author.
